# A cortical circuit for audio-visual predictions

**DOI:** 10.1038/s41593-021-00974-7

**Published:** 2021-12-02

**Authors:** Aleena R. Garner, Georg B. Keller

**Affiliations:** 1 Friedrich Miescher Institute for Biomedical Research, Basel, Switzerland; 2 Faculty of Natural Sciences, University of Basel, Basel, Switzerland

**Keywords:** Visual system, Learning and memory, Auditory system

## Abstract

Learned associations between stimuli in different sensory modalities can shape the way we perceive these stimuli. However, it is not well understood how these interactions are mediated or at what level of the processing hierarchy they occur. Here we describe a neural mechanism by which an auditory input can shape visual representations of behaviorally relevant stimuli through direct interactions between auditory and visual cortices in mice. We show that the association of an auditory stimulus with a visual stimulus in a behaviorally relevant context leads to experience-dependent suppression of visual responses in primary visual cortex (V1). Auditory cortex axons carry a mixture of auditory and retinotopically matched visual input to V1, and optogenetic stimulation of these axons selectively suppresses V1 neurons that are responsive to the associated visual stimulus after, but not before, learning. Our results suggest that cross-modal associations can be communicated by long-range cortical connections and that, with learning, these cross-modal connections function to suppress responses to predictable input.

## Abstract (web summary)

What you hear influences what you see. The authors show that experience with an audio-visual stimulus reshapes the input from auditory cortex to visual cortex, suppressing predictable visual input to amplify the unpredictable.

## Main

**Fig. 1:**
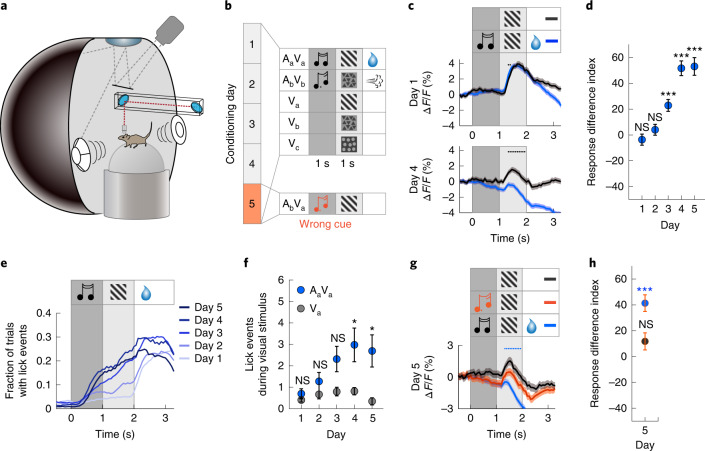
V1 responses are suppressed by an associated auditory cue. **a**, Schematic representation of the VR setup. **b**, Experimental paradigm. Over the course of five conditioning days, mice were exposed to auditory-cued visual stimuli (A_a_V_a_ and A_b_V_b_) that were reinforced, to the visual stimuli alone (V_a_ and V_b_) with no reinforcement, and to a control visual stimulus (V_c_) that was never paired with an auditory stimulus or reinforced. On day 5, mice were additionally exposed to a previously unexperienced audio-visual stimulus pair (A_b_V_a_). **c**, Average population responses of L2/3 V1 neurons for cued (A_a_V_a_, blue) and un-cued (V_a_, gray) visual stimulus presentations on day 1 (top) and day 4 (bottom) of conditioning. Traces and shading indicate mean ± s.e.m. across neurons. For **c**, **d**, **g** and **h**, days 1–4: *n* = 1,548 neurons from ten mice; day 5: *n* = 1,341 neurons from nine mice. Black dots indicate that traces are different during visual stimulation (*P* < 0.05, paired two-sided *t*-test; see Methods for detailed calculations). Here, and in subsequent figures, the dark gray bar indicates auditory stimulus presentation, and the light gray bar indicates visual stimulus presentation. **d**, Quantification of the difference in response for each conditioning day (response difference index) during the auditory-cued and un-cued visual stimulus presentations, normalized by the mean response during the un-cued visual stimulus on day 1 (V_a_− A_a_V_a_)/mean(V_a_). Asterisks indicate comparison to 0 difference using a two-sided rank-sum test. Days 1–5, respectively: *P* = 0.258, *P* = 0.183, *P* = 1.19 × 10^−6^, *P* = 4.77 × 10^−28^, *P* = 4.93 × 10^−15^. Here and in subsequent panels: *P < 0.05, **P < 0.01, ***P < 0.001. **e**, Anticipatory licking increases with conditioning day for A_a_V_a_. Traces indicate mean fraction of trials with lick events. For **e** and **f**, days 1–4: *n* = ten mice and day 5: *n* = nine mice. **f**, Anticipatory licking for A_a_V_a_ (blue) and V_a_ (gray) with conditioning as quantified by lick events during visual stimulus presentation. Dot plots and error bars indicate mean ± s.e.m. across mice. Asterisks indicate comparison between A_a_V_a_ and V_a_ trials using a two-sided rank-sum test. Days 1–5, respectively: *P* = 0.426, *P* = 0.308, *P* = 0.064, *P* = 0.045, *P* = 0.004. **g**, Mean population responses on day 5 on which a subset of trials consisted of previously unpaired stimuli (A_b_V_a_). The response during A_b_V_a_ (orange) was different from the response during A_a_V_a_ (blue) but not from the response during V_a_ (gray). Traces and shading indicate mean ± s.e.m. across neurons. Blue dots indicate that A_b_V_a_ and A_a_V_a_ curves are different (Methods). **h**, Quantification of the difference in responses in **g** (response difference index). The response during the visual stimulus of condition A_b_V_a_ is greater than that during condition A_a_V_a_ (blue with orange), *P* = 1.49 × 10^−16^, but not different from the response during V_a_ (gray with orange), *P* = 0.372. Dot plots and error bars indicate mean ± s.e.m. across neurons. Comparisons were made using a two-sided rank-sum test. NS, not significant.

Although experience-dependent, cross-modal phenomena between auditory and visual perception, such as the McGurk effect^1^, have long been recognized, the neural circuit mechanisms responsible for such interactions have remained elusive. Here we probe the function of the direct interactions between auditory and visual cortices on processing of visual stimuli. During audio-visual associative learning, auditory cortex (AuC) is thought to underlie multi-modal plasticity in visual cortex^2–4^. Auditory input is known to influence neural activity in V1 (refs. ^5–8^), and some of these cross-modal responses are thought to be driven by direct projections from AuC that target local inhibitory circuits in V1 (ref. ^9^). Although the computational role of these interactions remains unclear, we hypothesized that long-range cortical connections are shaped by experience and function to communicate memories of stimulus associations. Specifically, we investigated whether the utility of such cross-modal interactions could be to compute a comparison between expected, or predictable, and actual sensory experience. To do this, we used an audio-visual associative conditioning paradigm and quantified how cross-modal interactions shape neural responses in V1 over the course of learning. Mice explored a virtual environment in which they were exposed to sequentially paired presentations of auditory and visual stimuli. A virtual environment was used to enable simultaneous head-fixed optical physiology and experimental control of both visual and auditory input. Over the course of five conditioning sessions (approximately 45 min each on five consecutive days), mice were presented with pairings of a 1-s auditory cue (A) followed by a 1-s visual stimulus (V) (Fig. 1a, b). For each mouse, two pairs of an auditory cue and a visual stimulus were presented throughout conditioning (A_a_V_a_ and A_b_V_b_). The specific identities of stimuli used were counterbalanced across mice. To quantify the responses to the visual stimuli without a preceding auditory cue, we occasionally presented the normally cued visual stimuli alone (V_a_ and V_b_) and also presented a control visual stimulus (V_c_) that was never paired with an auditory cue. On day 5 of the conditioning paradigm, on a subset of trials, we additionally probed responses to an auditory cue and visual stimulus pairing that the mouse had previously not experienced (A_b_V_a_). All presentations were randomized with an inter-stimulus interval of between 4 s and 12 s (Methods).

**Extended Data Fig. 1:**
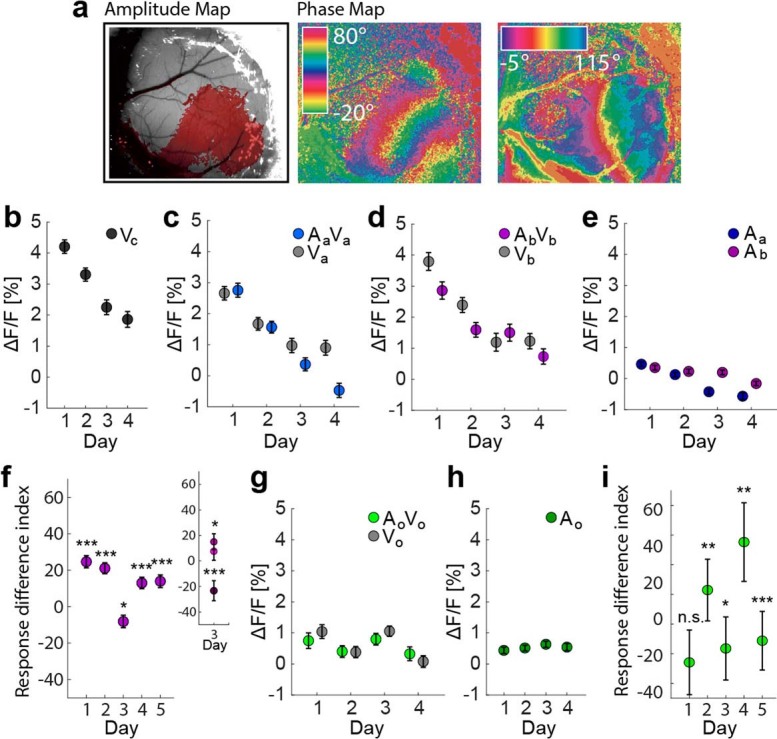
Mean V1 responses for aversive and unreinforced conditions. (**a**) Intrinsic signal optical imaging was performed on all mice before 2-photon imaging, n = 30 mice. Shown are data from one representative mouse. (**b**) Average population visual responses as a function of conditioning day to V_c_ (never paired), (**c**) visual responses to A_a_V_a_ (positive reinforcement, blue) and V_a_ (gray), (**d**) visual responses to A_b_V_b_ (negative reinforcement, pink) and V_b_ (gray), and (**e**) responses to the auditory cue, A_a_ (blue) and A_b_ (maroon). For **b - e** n = 1548 neurons from 10 mice. (**f**) Quantification of the difference in response for each conditioning day (Response difference index) during the auditory-cued and un-cued visual stimulus presentations, normalized by the mean response during the un-cued visual stimulus on day 1 (V_a_- A_a_V_a_)/ mean(V_a_). On day 3, the visual response to A_b_V_b_ was on average larger than that to V_b_ (see also panel **d**) resulting in a negative suppression. However, this effect was driven by a few outliers, which can be seen when the data is split into three epochs (inset). The negative suppression is only present in the 3^rd^ epoch of the day. Day 1 - 4: n = 1548 neurons from 10 mice; day 5: n = 1341 neurons from 9 mice. Asterisks indicate comparison to 0 difference using a two-sided rank-sum test. Here and in subsequent panels *: p < 0.05, **: p < 0.01, ***: p < 0.001. For detailed statistical analyses and exact p values see Supplementary Table 1. (**g**) Average population visual responses as a function of conditioning day when stimuli were not reinforced. A_o_V_o_ (no reinforcement, green) and V_o_ (gray), and (**h**) responses to the auditory cue A_o_ (dark green). For **g** and **h**, n = 496 neurons from 7 mice. Subscript o indicates an average across conditions a and b (that is A_a_V_a_ and A_b_ V_b_, V_a_ and V_b_, A_a_ and A_b_) because neither condition a or b was reinforced. (**i**) Quantification of the difference in response for each conditioning day (Response difference index) during the auditory-cued and un-cued visual stimulus presentations in the no reinforcement paradigm. Calculated as in panel **f**. Day 1 - 4: n = 496 neurons from 7 mice, day 5: n = 335 neurons from 5 mice. For **b - i**, dot plots represent mean and error bars represent SEM across neurons.

The behavioral relevance of visual stimuli is known to influence the dynamics of neural responses in V1 in paradigms in which the animal is exposed to the same stimuli over the course of days^10–12^. To test the influence of the behavioral relevance of the paired stimuli, we performed two variants of the conditioning paradigm in two groups of mice: one in which the paired stimuli were followed by appetitive or aversive reinforcements, and one in which the paired stimuli were not reinforced. In the reinforced variant, A_a_V_a_ was followed by a water reward and A_b_V_b_ by a mild air puff to the neck. Mice were neither required nor incentivized to perform differential behavior for paired and unpaired visual stimuli so that presentation of the visual stimulus alone was objectively neutral and not a reinforced stimulus on its own. Our aim was to prevent mice from consistently performing two distinct types of behavior for paired verses unpaired visual stimuli, which would confound the ability to analyze auditory-stimulus-specific effects. To monitor neural activity, 3 weeks before the conditioning experiments we injected an adeno-associated viral (AAV) vector expressing a genetically encoded calcium indicator (AAV2/1-EF1α-GCaMP6f) in right monocular V1. Throughout conditioning, mice were head-fixed on a spherical treadmill and free to locomote. Rotation of the treadmill was coupled to movement in a virtual tunnel displayed on a toroidal screen surrounding the mouse. The precise location of V1 in retinotopic coordinates was measured for all mice using optical imaging of intrinsic signals (Extended Data Fig. 1a). We recorded neural activity in layer 2/3 (L2/3) of V1 using two-photon calcium imaging. Visual stimuli were presented bilaterally in visual space matched to the retinotopic location of the two-photon imaging region. Auditory stimuli were presented through a speaker pair located symmetrically on either side of the mouse.

## Results

### Visual responses are suppressed by an associated auditory cue

**Extended Data Fig. 2:**
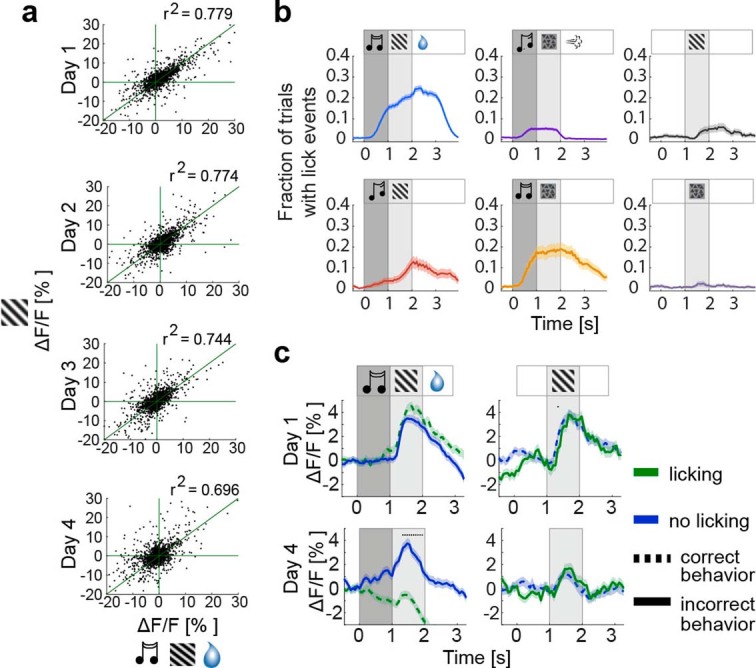
V1 response and licking dynamics. (**a**) The proportion of explained variance comparing responses during the visual stimulus presented alone, V_a_, and presented following the auditory cue, A_a_V_a_, decreases with conditioning day. r^2^ for the entire population of neurons is indicated on scatter plots; r^2^ per mouse mean ± SEM: day 1: 0.738 ± 0.051, day 4: 0.517 ± 0.091, p < 0.05 paired t-test, r values day 1 vs. day 4 comparison, n = 1548 neurons from 10 mice for **a**, **b**, and **d**. (**b**) Fraction of lick events (mean ± SEM) for stimulus conditions A_a_V_a_, A_b_V_b_, V_a_, A_a_V_b_, A_b_V_a_, V_b_, respectively on Day 5 of conditioning. n = 9 mice. (**c**). Average population responses of L2/3 V1 neurons (mean ± SEM) to A_a_V_a_ (left) and V_a_ (right) on day 1 (top) and day 4 (bottom) for trials during which mice licked (green) and failed to lick (blue). Dashed lines indicate correct licking preceding reward or correct withholding of licking preceding no reward during stimulus presentations. Solid lines indicate the converse (incorrect) licking behavior. Here and in subsequent figures, black dots indicate traces are different during visual stimulation (p < 0.05, paired two-sided t-test, see Methods for detailed calculation).

To first assess the effect of repeated exposure to a visual stimulus over the course of conditioning, we examined population responses to V_c_, which was never paired with an auditory cue or reinforced, and found a general decrease in responsiveness across days (Extended Data Fig. 1b). To test whether experience with audio-visual sequential pairings affected whether V1 responded differently to a visual stimulus, we first compared the average population responses to the auditory cue and visual stimulus pair that was followed by a reward (A_a_V_a_) to that of the same visual stimulus (V_a_) presented alone. We found that, on day 1 of conditioning, the two visual responses were similar (Fig. 1c). Analogous to V_c_, over the course of conditioning, the visual responses to both A_a_V_a_ and V_a_ decreased (Extended Data Fig. 1c). Interestingly, however, we found that the auditory cue preceding the paired visual stimulus resulted in an additional suppression of the visual response that increased with experience (Fig. 1c,d and Extended Data Fig. 1c). Furthermore, this suppression was most prominent for the auditory and visual stimuli followed by a water reward. For the audio-visual stimuli followed by an air puff (A_b_V_b_), we also observed a suppression of the visual response after the auditory cue; however, this suppression developed already on day 1 and was weaker and more variable than in the rewarded condition (Extended Data Fig. 1d,f). Additionally, the auditory cue itself resulted in a slight increase in V1 activity initially and a slight decrease in activity later in conditioning (Extended Data Fig. 1e). In mice that underwent the same pairing paradigm without any reinforcements, visual responses were smaller on average (Extended Data Fig. 1g), and the auditory cue did not result in a consistent suppression of the visual response (Extended Data Fig. 1g,i). Similar to reinforced conditioning, the auditory cue itself initially resulted in a slight increase in activity, but, unlike reinforced conditioning, this response did not change over time (Extended Data Fig. 1h). To investigate the mechanism of auditory-cue-driven suppression of visual responses, we focused subsequent analyses on the stimuli that were reinforced with a water reward. In addition to the experience-dependent auditory-cue-driven suppression, we also found that the visual responses to A_a_V_a_ and V_a_ de-correlated with experience (Extended Data Fig. 2a). Thus, experience with sequential audio-visual pairings can change the way V1 represents visual stimuli depending on the behavioral relevance of the stimuli.

**Extended Data Fig. 3:**
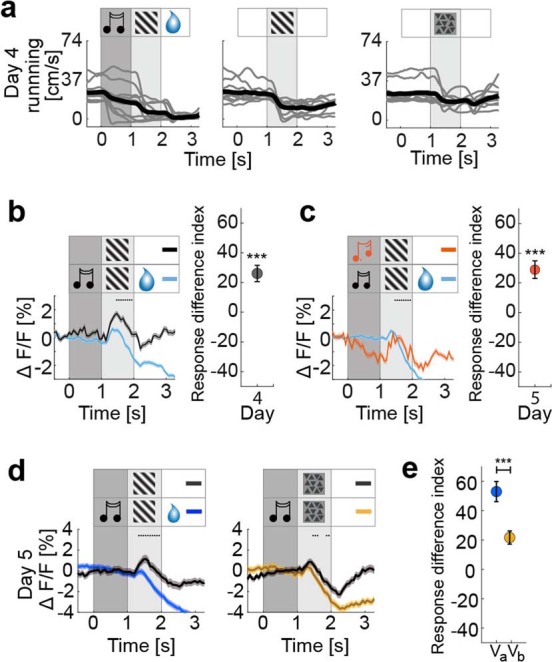
Running speed controls and specificity of suppression of A_a_ for V_a_. (**a**) Average running speeds during stimulus presentations (gray, each mouse; black, mean across mice), n = 10 mice, running speed before stimulus onset, 25.2 ± 2.9 cm/s and during visual stimulation, A_a_V_a_: 7.6 ± 2.4 cm/s, V_a_: 12.7 ± 1.6 cm/s, and V_c_: 18.1 ± 1.4 cm/s (mean ± SEM). (**b**) (Left) Average population responses for cued (A_a_V_a_, blue) and un-cued (V_a_, gray) visual stimulus presentations on day 4 of conditioning for running speed matched trials. Average speed and total number of trials included for A_a_V_a_: 9.0 ± 0.4 cm/s, 487 trials and for V_a_: 8.9 ± 0.5 cm/s, 126 trials. (Right) Response difference index. p = 5.41*10^−10^. Asterisk indicates comparison to 0 difference using a two-sided rank-sum test. n = 1548 neurons from 10 mice. Here and in subsequent panels *: p < 0.05, **: p < 0.01, ***: p < 0.001. For **b - e**, traces or filled circles indicate the mean and shading or error bars indicate SEM across neurons. (**c**) (Left) Average population responses of L2/3 V1 neurons for the previously paired cue (A_a_V_a_, blue) and previously un-paired cue (A_b_V_a_, orange) visual stimulus conditions on day 5 of conditioning for running speed matched trials. Average speed and total number of trials included for A_a_V_a_: 11.3 ± 0.4 cm/s, 857 trials and for A_b_V_a_: 10.6 ± 0.8 cm/s, 92 trials. (Right) Response difference index. p = 5.44*10^−14^. Asterisk indicates comparison to 0 difference using a two-sided rank-sum test. n = 1341 neurons from 9 mice. (**d**) Average population responses during visual stimulation for previously paired stimuli (left) following the cue (A_a_V_a_, blue) and un-cued (V_a_, gray) and for previously unpaired stimuli (right) following the same cue (A_a_V_b_, yellow) and un-cued (V_b_) visual stimulus presentations on day 5 of conditioning. Traces were baseline subtracted during the auditory cue (-667 - 0 ms before visual stimulus onset). For **d** and **e** n = 1341 neurons from 9 mice. (**e**) Comparison of response difference index for A_a_V_a_ and V_a_ (blue) verses A_a_V_b_ and V_b_ (yellow). p = 2.52*10^−4^. Asterisks indicate comparison between the response difference index for each condition using a two-sided rank-sum test.

Mice exhibited an appetitive conditioned behavioral response—anticipatory licking—in the reinforced paradigm (Extended Data Fig. 2b). To measure whether the licking response evolved on a time scale similar to that of the audio-visual suppression during conditioning, we quantified licking in anticipation of the water reward. Over the course of conditioning days, mice successively increased the number of licks made before reward delivery during the presentation of the auditory-cued visual stimulus, A_a_V_a_ (Fig. 1e). Although the presentation of the visual stimulus in the absence of the auditory cue, V_a_, also resulted in occasional licking, this response was much weaker (Fig. 1f). To test whether auditory-cue-driven suppression of visual responses was caused by a differential behavioral response during A_a_V_a_ and V_a_, we took advantage of the variability in licking behavior. Although mice exhibited an increased licking response to the auditory-cued visual stimulus, they also exhibited licking in a subset of non-cued visual stimulus trials (day 1: 26.8% ± 5.3% of trials and day 4: 39.9% ± 8.4% of trials, mean ± s.e.m.) and did not lick during a subset of the auditory-cued visual stimulus trials (day 1: 63.5% ± 9.2% of trials and day 4: 27.2% ± 9.4% of trials, mean ± s.e.m.). We could thus compare the responses in trials with and without licking for both conditions separately (Extended Data Fig. 2c). On day 1, we found no response difference induced by licking. On day 4, licking also did not result in a reduction of the response to the visual stimulus when presented alone, indicating that licking per se did not drive a suppression of the visual response. However, for the auditory-cued visual response, the suppression on day 4 was present only in trials in which the mouse exhibited anticipatory licks to the reward. Thus, after conditioning, the auditory cue only resulted in a suppression of the visual response when it was accompanied by a licking response. This suggests that mice must acknowledge presentation of the paired stimuli for the auditory cue to have a suppressive effect on the visual response. In parallel to the anticipatory licking responses, both auditory and visual stimuli induced a reduction in average running speed (Extended Data Fig. 3a), which is known to modulate visual responses^13–15^. However, auditory-cue-driven suppression was not explained by variance in running, as it was still present in speed-matched trials (Extended Data Fig. 3b,c). Thus, differences in running speed cannot account for the observed experience-dependent suppression of the visual responses by the auditory cue.

To determine whether suppression of the visual response developed specifically for the auditory cue paired with the visual stimulus, we presented previously unpaired auditory cue and visual stimulus pairings in a subset of the trials on day 5 of conditioning (A_b_V_a_). We found that suppression of the visual response was specific to the auditory cue with which the visual stimulus had been paired. There was no suppression when the visual stimulus was preceded by a different auditory cue than the one with which it had been associated, and the response to the visual stimulus after a different auditory cue, A_b_V_a_, was not different from the response to the visual stimulus alone, V_a_ (Fig. 1g,h and Extended Data Fig. 3c). Furthermore, suppression of the visual response after the auditory cue A_a_ was greater for the paired visual stimulus, V_a_, than for a previously unpaired visual stimulus, V_b_ (Extended Data Fig. 3d,e). In summary, we found that, in a behaviorally relevant context, the association of an auditory cue with a visual stimulus results in a stimulus-specific suppression of the visual response in L2/3 of V1.

### Auditory input to V1 is multi-modal and experience dependent

**Fig. 2:**
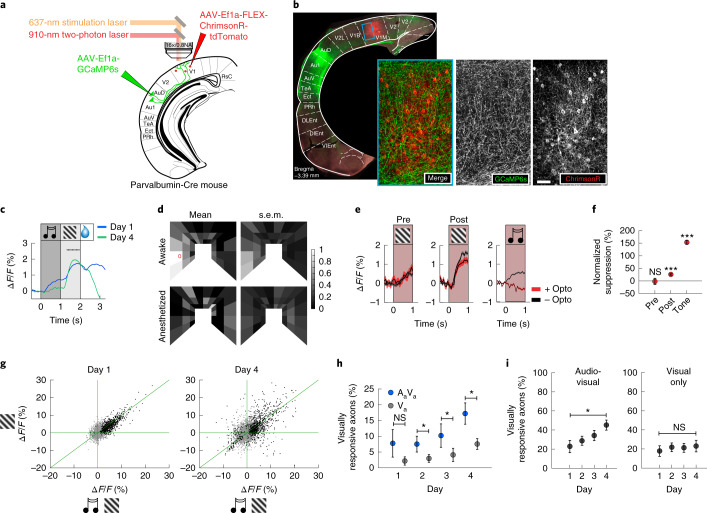
AuC sends experience-dependent audio-visual signals to V1. **a**, Schematic of injection sites referenced to atlas^50^. GCaMP6s injection in AuC and ChrimsonR-tdTomato injection in V1. **b**, Confocal histology image illustrating AuC axonal projections to V1 neurons (green) and V1 PV neurons (red) at the approximate imaging location. Insets show region marked by blue box in V1. Scale bar, 50 µm. **c**, AuC axons in V1 respond to the auditory cue and to the visual stimulus. Day 1: *n* = 21,076 axons from 20 mice and day 4: *n* = 19,486 axons from 19 mice. See also Extended Data Fig. 3c–e. Traces and shading represent mean and s.e.m., respectively, across axons. Black dots indicate that traces are different during visual stimulation (*P* < 0.05, paired two-sided *t*-test; see Methods for detailed calculations). **d**, Visual responses of AuC axons were mapped in a virtual corridor environment (Methods). Visual responses of AuC projection axons were retinotopically matched to the imaging location in V1 in awake mice (top, 4,305 axons in seven mice). The red circle marks the average peak location of visual responses of V1 neurons recorded in the same anatomical location and the same stimulation setup^51^. In anesthetized mice, visual responses were nearly absent (bottom, 991 axons in five mice). Left column, mean responses plotted as a function of location in visual space in the virtual corridor. Right column, corresponding s.e.m. Color scale is normalized to the peak response (1.1% Δ*F*/*F*). **e**, Inhibiting V1 locally by optogenetic excitation of PV-positive interneurons had no effect on visual responses before conditioning (left, 2,927 axons in seven mice) and a moderately suppressive effect after conditioning (middle, 3,857 axons in seven mice) but resulted in complete suppression of auditory responses (right, 4,130 axons in six mice). Red bar indicates laser illumination. Traces and shading represent mean and s.e.m., respectively, across axons. **f**, Normalized suppression quantified as the difference between the response to the stimulus with and without optogenetic inhibition, normalized by the mean response to the stimulus without inhibition. Pre: *n* = 2,927 axons from seven mice, *P* = 0.178; Post: *n* = 3,857 axons from seven mice, *P* = 1.58 × 10^−20^. Tone: *n* = 4,130 axons from six mice, *P* = 2.42 × 10^−176^. Asterisks indicate comparison to 0% suppression using a two-sided rank-sum test. Here and in subsequent panels: *P < 0.05, **P < 0.01, ***P < 0.001. Dot plots and error bars represent mean ± s.e.m. across axons. **g**, Average visual response of each axon to A_a_V_a_ plotted against the visual response to V_a_ on day 1 (left) and day 4 (right). Black data points are axons with a significant response to either visual stimulus condition. For **g**–**i**, day 1: *n* = 5,552 axons from eight mice, day 2: *n* = 4,697 axons from seven mice, day 3: *n* = 4,437 axons from seven mice and day 4: *n* = 4,336 axons from six mice. **h**, Fraction of visually responsive axons to A_a_V_a_ (blue) and V_a_ (gray) as a function of conditioning day. Comparisons were made using a paired two-sided *t*-test. For day 1–4, respectively, *P* = 0.133, *P* = 0.029, *P* = 0.020 and *P* = 0.011. For **h** and **i**, dot plots and error bars represent mean ± s.e.m. across axons. **i**, Left, fraction of visually responsive axons as a function of conditioning day in the audio-visual conditioning context. Right, For the same mice and axons, in a visual only context, the fraction of visually responsive axons did not change from day 1 to day 4. *P*^audio−visual^ = 0.020 and *P*^visual only^ = 0.536. Comparisons were made using an unpaired two-sided *t*-test. NS, not significant.

**Extended Data Fig. 4:**
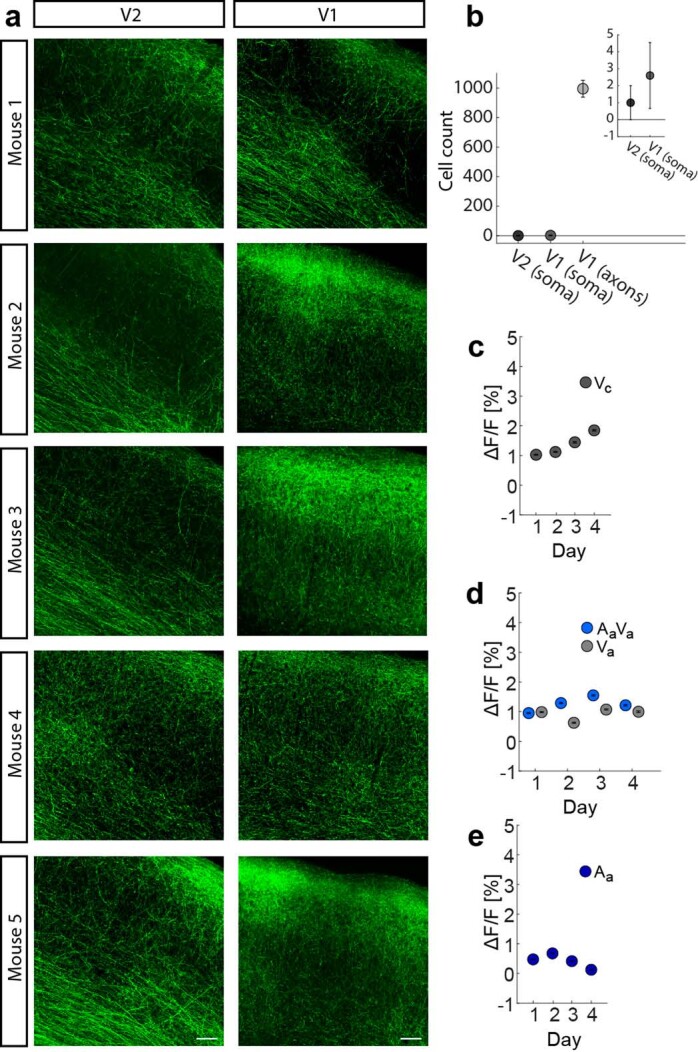
AuC injections label neurons in AuC. (**a**) Injection in AuC (see methods) to label projection axons (green). Z projection of confocal images shows approximately 656 ×656 x 32 um of secondary visual cortex (V2)(left) and V1 (right). Scale bar indicates 50 µm. For **a** and **b** soma counts from histology n = 5 mice. (**b**) Quantification of infected soma in V2 (left) and V1 (middle), and axons in V1 (right) after injection in AuC. Inset: same but scaled to range of soma numbers. Dot plots and error bars represent mean ± SEM across mice. (**c**) Average population visual responses as a function of conditioning day to V_c_ (never paired), (**d**) visual responses to A_a_V_a_ (positive reinforcement, blue) and V_a_ (gray), (**e**) and responses to the auditory cue, A_a_. For **c - e**, day 1: n = 5552 axons from 8 mice, day 2: n = 5097 axons from 8 mice, day 3: n = 5157 axons from 8 mice, and day 4: n = 4658 axons from 7 mice. For **c - e**, dot plots and error bars represent mean ± SEM across axons.

Visual and auditory cortices directly interact both anatomically and functionally^4,6,16–19^, resulting in responses to visual and auditory stimuli in both regions^2,18,20^. AuC projects directly to V1 in primates^19,21^ and rodents^22,23^, where it constitutes one of the densest inputs to V1, as quantified by rabies tracing in mice^24^. To test whether direct projections from AuC to V1 could contribute to the auditory-cued suppression of visual responses, we repeated the conditioning experiments in a cohort of mice in which we functionally imaged AuC axons in V1. We injected an AAV2/1-EF1α-GCaMP6s vector in AuC to express GCaMP6s in AuC neurons and implanted an imaging window over ipsilateral V1 to perform two-photon imaging of superficial AuC projection axons in V1 (Fig. 2a,b). We confirmed in postmortem histological analysis that the vast majority of the neurons labeled were in AuC and that the few neurons retrogradely labeled in V1 could not account for the number of axons that we recorded in V1 (Extended Data Fig. 4a,b).

Recording the activity of AuC axons in V1, we found that, early in conditioning, these carried both an auditory response and a visual response (Fig. 2c). Interestingly, the visual responses were larger than the auditory responses and, differently from responses in V1, increased slightly over the course of conditioning (Fig. 2c and Extended Data Fig. 4c,d). Conversely, the auditory responses in AuC axons, like the visual responses in V1, decreased across conditioning days (Fig. 2c and Extended Data Fig. 4e). Intrigued by the strength of the visual responses, we mapped the responses as a function of retinotopic location of the visual stimulus and found that they had receptive fields that matched the retinotopic location of the recording location in V1 (Fig. 2d, top). This is consistent with the interpretation that the responses after the visual stimulus onset in the paired presentation, A_a_V_a_, are likely visually driven and not delayed auditory responses or anticipatory motor responses. These visual responses were absent in anesthetized recordings (Fig. 2d, bottom), suggesting that the visual responses might arise from cortico-cortical top-down-like connections^25,26^. Given that visual cortex also projects to AuC^16,20^, it is possible that the source of the visual responses in AuC axons is inherited from retinotopically matched V1 neurons. To test this, we examined AuC axon responses while silencing activity in V1 locally. We used a mouse line expressing Cre in parvalbumin (PV)-positive interneurons^27^ and injected an AVV vector to express a Cre-dependent channelrhodopsin variant in V1 (AAV2/1-EF1α-DIO-ChrimsonR-tdTomato). We then quantified the effect of locally silencing V1 using optogenetic activation of PV interneurons while imaging the calcium responses in AuC axons (Methods). Surprisingly, we found that the inhibition of V1 activity was effective in suppressing auditory-evoked responses in the AuC axons but resulted in no suppression of visual responses before conditioning and only a small reduction after conditioning (Fig. 2e,f). The responsiveness of AuC projection axons to visual stimuli is consistent with previous work in awake mice showing that visually responsive neurons in AuC are predominantly found in layers 5 and 6 (ref. ^28^), which send collaterals to cortical targets, including V1 (ref. ^9^). However, the role of visual responses in AuC remains elusive. Our results show that AuC conveys a retinotopically matched visual signal to V1 largely independent of V1 activity. Such a signal could potentially function to inhibit the auditory-cued visual response in visual cortex. For AuC input to contribute to the experience-dependent suppression of auditory-cued visual responses, we would expect an experience-dependent change in the AuC axon responses over the course of conditioning. Congruently, we found that there was a decrease of similarity between axon visual responses to A_a_V_a_ and V_a_ between day 1 and day 4 of conditioning (Fig. 2g). In addition, we found that the fraction of visually responsive axons was greater when the visual stimulus followed the auditory cue (A_a_V_a_) than when presented alone (V_a_) (Fig. 2h). This result prompted us to examine differences in visual responsivity of AuC axons when mice were tasked with learning audio-visual associations compared to when they were similarly exposed only to visual stimuli. We, therefore, exposed the mice in our audio-visual conditioning context to a second context, over the same time course of conditioning, in which only visual stimuli were presented (Methods). We found that, although the overall fraction of visually responsive axons increased from day 1 to day 4 of conditioning in the audio-visual context (Fig. 2i, left), there was no change in the fraction of visually responsive axons from day 1 to day 4 in the visual-only context (Fig. 2i, right). Thus, AuC input to V1 exhibits an experience-dependent modulation of the visual response by the auditory cue.

### AuC-mediated suppression is stimulus and experience dependent

**Fig. 3:**
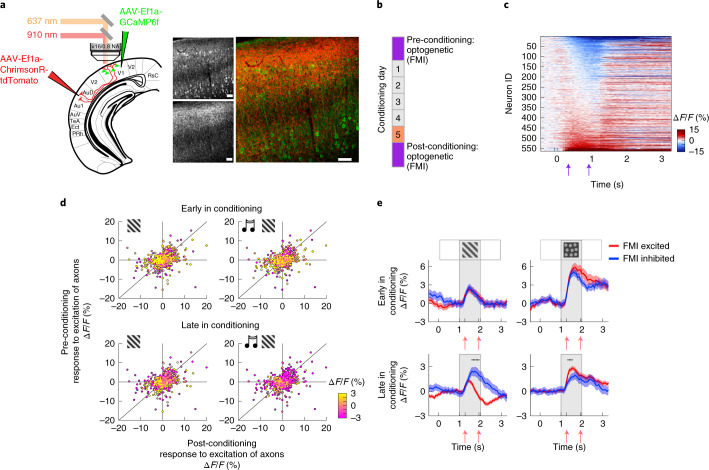
AuC selectively inhibits visually responsive neurons in V1. **a**, Left, schematic of injection sites referenced to atlas^50^. GCaMP6f injection in V1 and ChrimsonR-tdTomato injection in AuC. Right, confocal histology image illustrating AuC axons (bottom gray inset and red) and V1 neurons (top gray inset and green). Scale bars, 50 µm. **b**, Optical stimulation of AuC projection axons in V1 was performed to FMI of AuC input on V1 neurons 1 d before and 1 d after the 5-s conditioning paradigm. **c**, V1 neuron responses to pre-conditioning optogenetic stimulation of AuC axons sorted by strength of response. Purple arrows indicate the window over which response was averaged to generate FMI response values in **d**. For **c** and **d**, *n* = 563 neurons from five mice. **d**, The response of each V1 neuron to optogenetic stimulation of AuC axons (FMI) before conditioning plotted against the response after conditioning. Color indicates the visual response of each neuron to V_a_ (left) or A_a_V_a_ (right), early (top) and late (bottom) in conditioning. **e**, Visual responses of neurons inhibited (blue) or excited (red) by optogenetic excitation of AuC axons (FMI) to V_a_ (left) and V_c_ (right), early (top) and late (bottom) in conditioning. Colored arrows indicate the window over which response was averaged for individual neurons to calculate the visual response value plotted in **d**. Early in conditioning refers to the first exposure to stimuli, which occurred on the pre-FMI day using visual stimulus trials without optogenetic stimulation. *n* = 563 neurons, 257 FMI inhibited, from five mice. Late in conditioning refers to an average of visual responses from days 3 and 4 of the conditioning paradigm (see also Extended Data Fig. 4d,f). *n* = 1,548 neurons, 482 inhibited, from ten mice. Traces indicate the mean, and shading represents the s.e.m. across neurons. Black dots indicate that traces are different during visual stimulation (*P* < 0.05, paired two-sided *t*-test; see Methods for detailed calculations).

**Extended Data Fig. 5:**
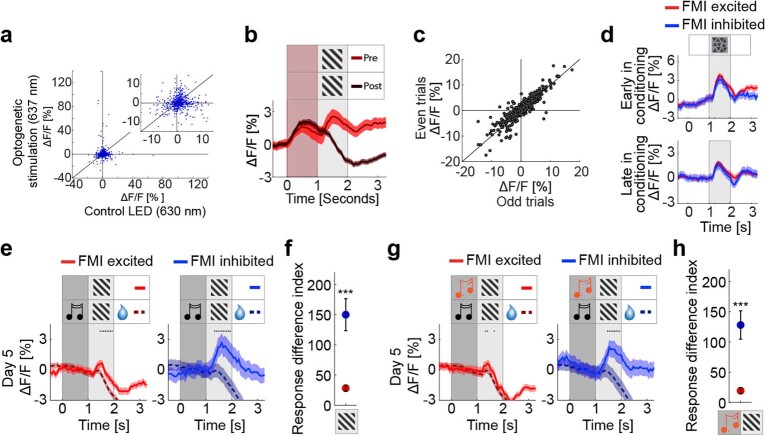
Functional Mapping of Influence (FMI) controls. (**a**) Population responses to optogenetic stimulation (y-axis) compared to sham stimulation (x-axis) show no correlation. r = -0.016, p = 0.71. For **a** and **c**, n = 563 neurons from 5 mice. (**b**) The average population response of V1 soma to optogenetic stimulation of AuC axons pre- (light red) and post- (dark red) conditioning and the average response across all visual stimuli, V_a_, V_b_, and V_c_ (indicated by grating icon). Pre: n = 563 neurons from 5 mice, Post n = 1548 neurons from 10 mice. For **b**, **d**, **e**, and **g**, traces represent the mean and shading represents SEM across neurons. (**c**) The response of all V1 neurons to optogenetic stimulation of AuC axons on even numbered trials plotted against the response on odd numbered trials. Correlation coefficient calculated using Pearson’s R. r = 0.933, p = 6.51*10^−252^. (**d**) Visual responses of neurons inhibited (blue) or excited (red) by optogenetic excitation of AuC axons (FMI) to V_b_ early (top) and late (bottom) in conditioning. Early in conditioning refers to first exposure to stimuli, which occurred on the pre-FMI day using visual stimulus trials without optogenetic stimulation. n = 563 neurons, 257 FMI inhibited, from 5 mice. Late in conditioning refers to an average of visual responses from days 3 and 4 of the conditioning paradigm. n = 1548 neurons, 482 inhibited from 10 mice. (**e**) Average population responses of V1 neurons excited (reds, left) and inhibited (blues, right) by AuC stimulation to V_a_ (solid trace) and A_a_V_a_ (dashed trace) presentations on conditioning day 5. For **e** - **h**: n = 1341 total, 927 excited, and 414 inhibited neurons from 9 mice. (**f**) Response difference index for data shown in **d**. p = 8.67 *10^−13^. Comparison between excited and inhibited neurons using a rank-sum test. For **f** and **h**, dot plots and error bars represent mean ± SEM across neurons. Here and in subsequent panels *: p < 0.05, **: p < 0.01, ***: p < 0.001; for all statistical analyses and exact p values see Supplementary Table 1. (**g**) Average population responses of V1 neurons excited (reds, left) and inhibited (blues, right) by AuC stimulation to A_b_V_a_ (solid trace) and A_a_V_a_ (dashed trace) presentations on conditioning day 5. (**h**) Response difference index for data shown in **f**. p = 1.10*10^−8^ Comparison between excited and inhibited neurons using a rank-sum test.

**Extended Data Fig. 6:**
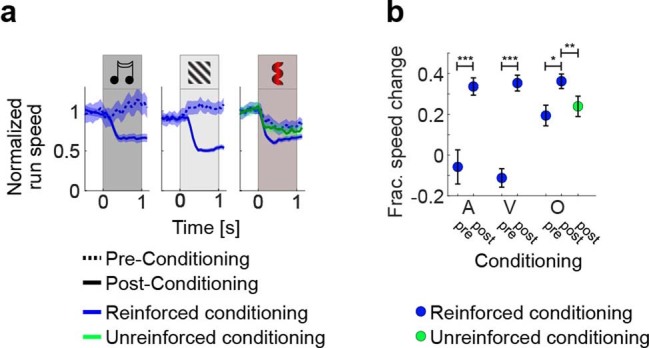
Activation of AuC has an experience-dependent influence on behavior. (**a**) Change in running speed induced by the auditory cue (left), the visual stimulus (middle), and the optogenetic activation of AuC axons in V1 (right), pre (dashed) and post conditioning (solid). The behavioral response to all three stimuli increases with conditioning. The behavioral response to the AuC axon stimulation was also larger in reinforced (n = 5) compared to unreinforced (n = 7) mice. Traces and shading represent mean ± SEM, respectively. (**b**) Comparison of the fraction of speed change pre vs. post conditioning. Comparison between pre and post conditioning or between reinforced (n = 5) and unreinforced (n = 7) conditioning using a rank-sum test. Dot plots and error bars represent mean ± SEM, respectively, across trials. p^A^ = 4.38*10^−7^, p^V^ = 7.71*10^−13^, p^O^ = 0.017, p^rein. vs unrein.^ = 0.004. *: p < 0.05, **: p < 0.01, ***: p < 0.001.

AuC input could functionally suppress the auditory-cued visual responses either by global suppression, independent of stimulus preference of neurons in V1, or by specific suppression of the neurons responsive to the visual stimulus paired with an auditory cue. Additionally, in either case, given that the audio-mediated suppressive effects that we observe in V1 are experience dependent, we also hypothesized a suppressive action of AuC input that would be learned with experience. To test if the AuC input to V1 could function as either a global or a functionally specific suppressive input, we used an experimental paradigm in which we mapped the functional influence of AuC input on V1 neurons before and after conditioning. We injected a vector expressing a channelrhodopsin variant (AAV2/1-EF1α-ChrimsonR-tdTomato) in AuC and a vector expressing GCaMP6f (AAV2/1-EF1α-GCaMP6f) in V1 (Fig. 3a). This allowed us to functionally map the influence (FMI) of the AuC axon stimulation on neural responses of L2/3 V1 neurons or, in other words, ‘tag’ V1 neurons based on how they respond to AuC axon stimulation before and after conditioning. We used a 1-s pulse of a 637-nm laser to activate the ChrimsonR in the imaging region during two-photon imaging (Methods). As the stimulation occurred optically coaxial with the two-photon imaging, the mouse’s eyes were shielded from stimulation light by the imaging cone. To control for a putative effect of the stimulation light directly driving a visual response, we also performed sham stimulations with a second light source diffusely illuminating the head of the mouse outside of the imaging cone. Stimulation of the AuC axons resulted in a variety of responses in V1 (Fig. 3c). In unconditioned mice, 37.7% ± 8.2% of neurons were responsive to AuC axon stimulation, and, of these, 48.4% ± 20.1% were inhibited (*n* = 5 mice). In conditioned mice, 35.4% ± 7.0% of neurons were responsive to AuC axon stimulation, and, of these, 30.6% ± 11.1% were inhibited (*n* = 10 mice). Although we also observed a response to the sham stimulation, we found no correlation between the response to AuC axon stimulation and sham stimulation (Extended Data Fig. 5a), indicating that the response to the optogenetic stimulation of the AuC axons cannot be explained by a visual response. We then examined if an experience-dependent alteration of the connection from AuC to V1 existed in the form of a difference in the pattern of activation induced in V1 by the AuC stimulation before and after audio-visual experience. We tested this by functionally mapping the influence of AuC axon stimulation in the same L2/3 V1 neurons before and after conditioning (Fig. 3b). This allowed us to determine whether a relationship existed between the responses of a neuron to sensory stimulation (that is, V_a_ and A_a_V_a_) and to the artificial activation of AuC projection axons and if there was an experience-dependent change in the influence of the AuC input on V1. Although visual responses decreased in general over the course of conditioning, the average V1 population response to artificial AuC axon activation remained similar before and after conditioning (Extended Data Fig. 5b). Plotting the response to the artificial AuC stimulation for every V1 neuron before conditioning against the response after conditioning revealed a variety of learning-related changes that were larger than those expected simply from response variability to the stimulation on a trial-by-trial basis (Fig. 3d and Extended Data Fig. 5c). If, with experience, the AuC input to V1 selectively targets V1 neurons responsive to the visual stimulus V_a_, which was paired with auditory cue A_a_, we would expect V_a_-responsive neurons to be selectively inhibited by the AuC stimulation. To examine this, we color-coded the response of each neuron to V_a_ and A_a_V_a_ early and late in conditioning on scatter plots of their responses to the AuC axon stimulation before and after conditioning (Fig. 3d). We found that, early in conditioning, no correlation existed between responses to the visual stimulus and responses to AuC axon stimulation. However, late in conditioning, neurons with the strongest excitatory responses to the visual stimulus tended to cluster in the lower-left quadrant of the scatter plot, meaning that the neurons that were functionally inhibited by the stimulation of AuC axons showed the strongest responses to V_a_. Moreover, the visual responses of these neurons were reduced in the A_a_V_a_ condition. To quantify this tendency and examine the stimulus specificity of AuC axon stimulation effects, we split V1 neurons into those inhibited by and those excited by AuC axon stimulation and compared visual responses of these populations. Neurons with a decrease in fluorescence during AuC axon stimulation were classified as inhibited and those with an increase as excited. This definition allowed inclusion of all neurons in the analysis. Although early in conditioning no difference existed between the mean visual responses of neurons either excited or inhibited by AuC axon stimulation, after conditioning the neurons inhibited by AuC axon stimulation exhibited larger responses specifically to V_a_ but not to V_c_ (Fig. 3e). Consistent with the result that the auditory cue A_a_ leads to a specific suppression of the neurons responsive to the visual stimulus V_a_, we also found no difference in the response to V_b_ between the neurons that were excited by the AuC axon stimulation and those that were inhibited (Extended Data Fig. 5d). Note that this result likely critically hinges on the different time courses and magnitudes of auditory-driven suppression of visual responses in appetitive and aversive learning. Importantly, the population of AuC-inhibited neurons carried most of the effect of the experience-dependent auditory-cue-driven suppression of the visual response to V_a_ (Extended Data Fig. 5e,f) and the largest recovery of the visual response after the previously unpaired auditory cue (Extended Data Fig. 5g,h). These results are consistent with a specific targeting of the functional inhibition to neurons receiving the strongest drive from the visual stimulus that was paired with the auditory cue. Thus, experience reshapes the influence of the long-range cortical connections between AuC and V1 to suppress responses to visual stimuli that the mouse learns to predict from auditory cues. To test whether this experience-dependent change of the AuC-to-V1 connection was involved in experience-dependent changes in behavior, we compared the change in running speed induced by the artificial activation of AuC axons before and after conditioning (Extended Data Fig. 6). With experience in the conditioning paradigm, mice exhibited an increase in the reduction of running speed upon presentation of the auditory cue or the visual stimulus. We observed a similar increase in the reduction of running speed triggered by the activation of the AuC axons. This is consistent with the interpretation that the experience-dependent change in the connection from AuC to V1 is behaviorally relevant.

## Discussion

**Extended Data Fig. 7:**
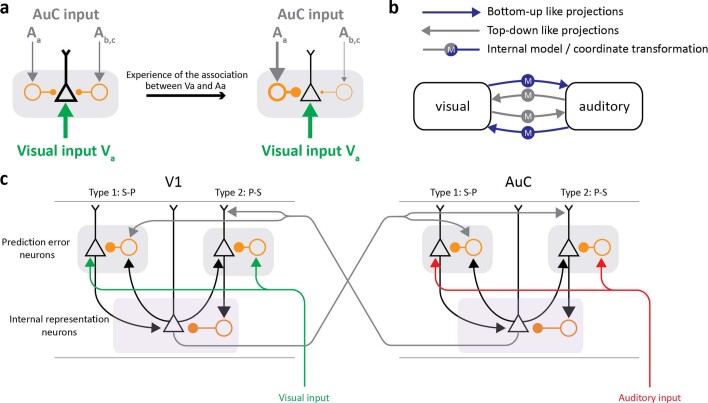
A conceptual model for audio-visual interactions. (**a**) Our results demonstrate that with experience, the top-down input from AuC to V1 rearranges to target the layer 2/3 neurons in V1 responsive to V_a_ for suppression. This is consistent with a cross-modal suppression of predictable bottom-up input in V1. (**b**) Given that the interaction between AuC and V1 is not hierarchical, our results suggest that predictive processing can be expanded to non-hierarchical interactions in cortex. This could be achieved, for example, as follows: V1 and AuC mutually exchange predictions through top-down like projections and in return receive prediction errors through bottom-up like projections. See also^38^ for an extended discussion of non-hierarchical predictive processing. (**c**) More specifically, the cortical circuit for predictive processing^38^ can be directly expanded to lateral interactions between AuC and V1 as described in the following. Please note, this is an attempt at integrating our results with previous work on cortical circuits for predictive processing, and not meant as a summary of our results. For simplicity, only the exchange of predictive top-down like signals is shown. Bottom-up visual input is compared to top-down predictions of visual input from AuC in prediction error neurons in V1. Our results are consistent with the responses of such prediction error neurons in layer 2/3. The model postulates that audio-visual integration then occurs by virtue of internal representation neurons integrating over these prediction error responses. Identifying internal representation neurons will be key to further validating this model and will likely hinge on having genetic access to the functionally identified prediction error neurons we describe here.

In summary, we found that the association of an auditory cue with a visual stimulus results in an experience-dependent suppression of the visual response in L2/3 V1 neurons that is specific to the paired association (Extended Data Fig. 7a). Although auditory modulation of visual cortex likely occurs via multiple pathways^2^, one of the mechanisms that contributes to this experience-dependent suppression of predictable visual stimulation is direct input from AuC. With experience, the functional influence of AuC input changes to selectively target the L2/3 V1 neurons responsive to the paired visual stimulus for inhibition. This inhibition is likely mediated by local inhibitory neurons that are recruited by AuC input^9,29,30^. Interestingly, most input from AuC to V1 appears to be a visually driven signal that is largely independent of activity in V1. Such a visual input to AuC could originate in postrhinal cortex^31^ or multisensory thalamic regions, such as the lateral posterior thalamic nucleus^32,33^. This architecture of parallel processing streams culminating in a cross-stream prediction is a biological substrate reminiscent of recent advances in machine learning that have enabled self-supervised learning^34–37^. As the AuC input functions to suppress predictable visual input, these interactions are well described by the framework of predictive processing. The predictive processing model postulates that prediction error neurons (thought to be in layer 2/3) compute a comparison of bottom-up sensory input and top-down prediction. In this model, positive prediction error responses signal more bottom-up input than predicted by top-down input. The simplest way to compute such a prediction error would be to subtract the top-down prediction from the bottom-up input. As bottom-up sensory signals become predictable, top-down input acts to suppress it. Our results can be integrated in a circuit model for hierarchical predictive processing in visual cortex^38^ and provide direct evidence for the idea that predictive processing can be expanded to non-hierarchical cross-modal interactions (Extended Data Fig. 7b,c). Similarly, long-range cortico-cortical interactions are also thought to contribute to the suppression of predictable sound associated with movement^39,40^. Additionally, we found that a learned behavioral response to the auditory-cued visual stimulus was necessary for visual suppression, a result consistent with previous work showing a correlation between experience-dependent changes in V1 responses and behavioral performance during appetitive learning but not passive viewing^12^. In primary AuC, appetitive and aversive conditioning have been shown, respectively, to lead to a decrease and an increase in response amplitude to a conditioned stimulus^41^. It is, therefore, possible that the weaker and more variable suppression in V1 during aversive conditioning is a result of the combination of an enhancement of the aversive conditioned stimulus response and a suppression resulting from the auditory-cue-driven predictability of the visual stimulus. Concordantly, given the lack of reliable auditory-cue-driven suppression of visual responses in mice for which stimuli are not reinforced, the degree of neural suppression might also be dependent on an animal’s subjective value of the stimuli, which is known to modulate neural responses^42,43^. Our results also support the idea that cortical circuits are shaped by experience to store cross-modal associations and, thereby, contribute to memory storage in sensory cortex^44–46^. Moreover, blocking of the formation of an association of a stimulus with a reinforcement can occur when two conditioned stimuli are used as predictors^47,48^. Because the auditory cue is predictive of reinforcements in our study, suppression of the visual response might be a mechanism of blocking. An associative memory trace is often considered to reside in higher association areas that receive convergent input from lower sensory areas. An alternative mechanism for such a trace is the synaptic change that combines or redirects information flow between long-range sensory projections and local sensory areas. We show that cross-modal learning can shape and redefine representational patterns of sensory stimuli through the interaction of long-range input with local circuits. Thus, long-range cross-modal interactions can shape representations of the sensory world, endowing early sensory cortex with a mnemonic capacity^7,49^ that functions to make cross-modal predictions.

## Methods

### Animals

All animal procedures were approved by and carried out in accordance with guidelines of the Veterinary Department of the Canton Basel-Stadt, Switzerland. C57BL/6 and PV-Cre mice, female and male, between the ages of 3 and 4 months and group-housed by gender were used in our studies. Mice were housed on a 12-h light/dark cycle in cages with horizontal running wheels at an ambient temperature of between 20 °C and 25 °C and humidity between 40% and 60%.

### Surgeries

Surgeries were performed as described previously^52^. In brief, mice were anesthetized using a mix of fentanyl (0.05 mg kg^−1^), medetomidine (0.5 mg kg^−1^) and midazolam (5 mg kg^−1^). A craniotomy of either 5 mm or 3 mm in diameter was made over V1; a glass coverslip was super-glued in place; and a custom-machined stainless steel head bar was implanted.

### AAV injections

Injections consisted of 100–250 nl of AAV vector with a titer in the range of 10^12^–10^14^ genome copies per ml. The coordinates of the injections in V1 were 2.7–2.8 mm lateral from the midline and 2.8–3.0 mm posterior from bregma. For AuC injections, the coordinates were 4.4 mm lateral from the midline and 2.6–2.8 mm posterior from bregma, and the injection pipette was rotated to be perpendicular to the brain surface. For somatic imaging in V1, we used AAV2/1-EF1α-GCaMP6f for V1 PV-Cre excitation; for FMI, we used AAV2/1-EF1α-ChrimsonR-tdTomato^53^; and, for AuC axon imaging, we used AAV2/1-EF1α-GCaMP6s^54^.

### Histology

For postmortem histological analyses, mice were transcardially perfused with 4% paraformaldehyde in PBS. Brains were isolated and maintained in 4% paraformaldehyde at 4 °C on a shaker overnight. The fixed tissue was then rinsed with PBS and sectioned into 70-µm- or 100-µm-thick slices using a vibratome. Sections were mounted and sealed with DAPI ProLong mounting medium. Sections for all mice were imaged using a Zeiss AxioScan.Z1 slide scanner at ×10 magnification (Zeiss Zen blue edition software). All images used for quantification of the number of neurons expressing GCaMP were acquired at ×20 magnification, 5-µm step, *z*-stack images using a confocal microscope (VisiView version 3.3 software). Atlas overlays for histological images were adapted from ref. ^50^. Atlas images were first aligned to both rhinal fissures and the external capsule of coronal sections, and, subsequently, the thickness of the cortex was adjusted to fit each individual mouse. Confocal ex vivo histology images were acquired for all mice.

### Quantification of AAV spread

Injections of AAV2/1-EF1α-GCaMP6s-WPRE in AuC for axonal imaging in V1 also result in axonal uptake and expression in V1 neurons that project to AuC. To quantify what fraction of the axons in V1 could come from retrogradely labeled V1 neurons, we used a separate set of five mice for histological quantification. Mice were injected with AAV2/1-EF1α-GCaMP6s-WPRE in AuC and sacrificed for histological analysis time-matched to the start of the imaging experiments. We performed a histological quantification using confocal images of fixed tissue in a region corresponding to the location of our two-photon imaging window. We then quantified the number of neurons per slice volume (656 µm × 656 µm × 32 µm). We found infected neurons in V1 in two of five mice with a mean ± s.e.m. across mice of 2.6 ± 1.9 neurons and five infected neurons in one of five mice in secondary visual areas (1 ± 1, mean ± s.e.m. across mice) (Extended Data Fig. 2a,b). Given that the number of axons we were able to image in V1 in a volume of 200 µm × 200 µm × 40 µm was more than two orders of magnitude larger (day 1: 1,054.8 ± 117.8, day 2: 893.2 ± 91.6, day 3: 1,008.2 ± 121.9 and day 4: 1,025.6 ± 130.0; mean ± s.e.m.), retrogradely labeled V1 neurons are unlikely to account for a substantial fraction of the axons recorded in V1. Note that the comparison by volume is not entirely straightforward as one would need to estimate the average fraction of total V1 volume that the axon of a given V1 neuron would be visible in. However, additionally arguing against a contamination by axons of V1 neurons is the fact that expression levels in retrogradely labeled neurons tend to be far lower than at the primary injection site^55^. Thus, although we cannot exclude that some of the axons in our dataset originated from retrogradely labeled V1 neurons, the vast majority of them were likely AuC projection axons.

### Two-photon imaging

Functional imaging of GCaMP6-expressing neurons was performed using a modified Thorlabs B-Scope. The illumination source for two-photon imaging was a femtosecond infrared laser (Spectra-Physics) tuned to a wavelength of 910 nm. A 12-kHz resonance scanner (Cambridge Technology) was used for line scanning to acquire data at a frame rate of 60 Hz at a resolution of 400 × 750 pixels. In addition, we used a piezo actuator (Physik Instrumente) to acquire images at four different depths by moving the objective (Nikon ×16, 0.8 NA) in 15-µm steps between frames, thereby reducing the effective frame rate per imaging plane to 15 Hz.

### Optogenetic stimulation during two-photon imaging

The methods for simultaneous two-photon imaging and optogenetic stimulation were described previously^24,56^. In brief, the illumination source for the ChrimsonR stimulation was a switchable 637-nm laser (OBIS, Coherent) used at an average power of 11 mW and triggered using a TTL pulse. A dichroic mirror (ZT775sp-2p, Chroma) was used to combine two-photon and optogenetic stimulation light, and a long-pass dichroic mirror (F38-555SG, Semrock) was used to filter GCaMP6 emission from illumination light. To prevent stimulation light artifacts, the 637-nm laser was synchronized to the turnaround times of the resonant scanner when data were not acquired. To reduce the influence of ringing artifacts in the amplifier, signals were digitally band-pass filtered at 80 MHz using a 1.6-GHz digitizer (NI-5772, National Instruments) and an FPGA (PXIe-7965, National Instruments) to implement a digital Fourier filter.

### Conditioning paradigm

#### Mice

Mice were handled by the experimenter every day for at least 1 week before being introduced to the virtual reality (VR). Water restriction began 1 week before the start of experiments in which a water reward was delivered, and mice received 1 ml of water per day. Three to five days before the experiment, mice were exposed and habituated to head fixation in the VR and rewarded with sunflower seeds after each exposure period. Mice were considered habituated when they voluntarily walked onto the experimenter’s hand and did not resist head fixation. During experiments, mice received supplemental water after conditioning if they had not consumed at least 1 ml in water rewards. Mice were monitored to ensure they maintained at least 80% of their original body weight. For V1 soma imaging, one cohort of five mice underwent optogenetic experimentation in the VR context on day 1, followed by 5 d of conditioning, followed by a final day of optogenetics. A second cohort of five mice had optogenetic experimentation after only 5 d of conditioning. For AuC axon imaging, 20 mice were conditioned for 4 d. One mouse was removed from the analysis on day 4 owing to insufficient image registration. Of these mice, eight were PV-Cre and were also used for optogenetic and visual-context-only experiments.

#### Stimuli

Auditory stimuli consisted of either 16.1-kHz or 10.5-kHz pure tones presented at approximately 65 dB SPL^29^. The three visual stimuli used were a sinusoidal grating, a geometric pattern of triangles and a geometric pattern of ovals. One of the associated stimuli (a and b) was always the grating, but the pairing of the stimuli was otherwise randomized and counterbalanced across animals. For paired conditions, the auditory stimulus was 1 s in duration, followed immediately by a visual stimulus 1 s in duration, followed immediately by a reinforcement: a—water reward, b—air puff. For visual-stimulus-only conditions, the visual stimulus was presented for 1 s and never reinforced. Approximately 25% of trials were the V_x_ condition during the first four conditioning days (day 1, V_a_: 24.5% ± 0.2%) and ~14% of trials on day 5 (V_a_: 13.8% ± 0.5%). The occurrence of V_c_ as a fraction of all un-cued visual stimulus trials was day 1: 50.1% ± 0.3 % and day 5: 33.9% ± 0.5%. On day 5, A_b_V_a_ occurred for ~14% of all cued visual stimulus trials (A_b_V_a_: 13.8 ± 0.6). Values reported are mean ± s.e.m. For axonal imaging, the visual-only paradigm was performed 1 d before and after conditioning as well as after the audio-visual paradigm on conditioning days (Fig. 2i). Stimuli consisted of full field grating presentations of eight orientations with a stimulus duration of 2 s and a gray (mean-luminance) inter-stimulus interval of 3 s. Optogenetic stimulation of AuC axons for FMI experiments was performed 1 d before and 1 d after the conditioning paradigm as described above. Stimuli were also presented occasionally on the same day as optogenetic stimulation for a couple of reasons. First, we wanted to obtain a relative measure of V1 neuron responsivity to natural visual stimulation and to artificial optogenetic stimulation of AuC axon input on the same day. This allowed us to control for whether neurons were different in their excitability in general before versus after conditioning or showed more specific changes in their responsiveness to visual stimuli.

#### VR

Mice were head-fixed and free to locomote on an air-supported polysterene ball. A virtual tunnel designed with low-contrast gray checkered walls was projected onto a toroidal screen surrounding the mouse and yoked to linear displacement of the ball. From the mouse’s perspective, the screen encompassed a visual field of approximately 240° horizontally and 100° vertically. One speaker was placed on the left side and one on the right side of the VR for presentation of auditory stimuli. The VR system was otherwise constructed as described previously^52^. A water spout was placed in front of mice, and licking was detected using a custom-made electrical circuit in which a mouse closes the circuit whenever its tongue contacts the metal spout or water droplet^57^. The resulting voltage was thresholded to calculate licking events.

### Image analysis

Regions of interest (ROIs) for soma were obtained using custom semi-automated image segmentation software. ROIs for axons were obtained in an automated process as previously described in Mukamel et. al.^58^ using a combination of principal and independent component analysis and image segmentation modified in-house. Fluorescence traces across time were then calculated as the mean pixel value in each ROI per frame. Δ*F*/*F* was calculated using median-normalized traces and filtered as described previously^59^. For axonal imaging, data came from the same location in the brain using blood vessel patterns for alignment, but individual axons were not matched across imaging time points.

### Data analysis

Data analysis was performed using custom-written MATLAB (MathWorks) code. To quantify differences between response curves during visual stimulation (Figs. 1c,g, 2c and 3e and Extended Data Figs. 2c, 3b,c,d and 5d,e g), Δ*F*/*F* was compared in a response time window (11 frames, 267−1,000 ms after visual stimulus onset) with a baseline subtraction during auditory stimulation (mean activity in a window preceding visual stimulus onset, 10 frames, −667 ms to 0 ms) bin by bin for one-frame (66.7-ms) time bins using a paired *t*-test (*P* < 0.05). Dots above response curves indicate significant difference for at least three consecutive bins. For quantification of responses during visual, auditory, optogenetic or sham stimulation, Δ*F*/*F* was averaged over the response time window (11 frames, 267−1,000 ms after stimulus onset) and baseline subtracted (mean activity in a window preceding stimulus onset, ten frames, −667 ms to 0 ms) (Figs. 1d,h, 2g and 3d,e and Extended Data Figs. 1b–i, 2a, 3b,c,e, 4c–e and 5a,c f,h). Mean neural activity is an average across trials and neurons. Mean behavioral data are an average across trials and mice. Licking and running were quantified during the response time window (Fig. 1f and Extended Data Figs. 2c and 3b,c). For quantification of visually responsive axons (Fig. 2g–i), Δ*F*/*F* during the response time window was compared to Δ*F*/*F* during the baseline window. Normalized suppression of AuC axons was quantified as the difference between the response to the stimulus with and without optical stimulation of V1 PV neurons, normalized by the mean response to the stimulus without optical stimulation (Fig. 2f). The response difference index was computed by subtracting the response during the visual stimulus after the auditory cue (A_a,b,o_V_a,b,o_) from that during the visual stimulus presented alone (V_a,b,o_) (Fig. 1d,h and Extended Data Figs. 1f,i, 3b,e and 5f), the visual stimulus after the paired cue (A_a_V_a_) from that during the unpaired cue (A_b_V_a_) (Fig. 1h and Extended Data Fig. 3c and 5h) or the visual stimulus after the unpaired cue (A_b_V_a_) from that during the visual stimulus alone (V_a,b,o_) (Fig. 1h) or (A_a_V_b_) from (V_b_) (Extended Data Fig. 3e) and normalized to the mean visual response alone (V_a,b,o_) on day 1 of conditioning. Note that we used a subtractive measure normalized by day 1 responses to avoid division by 0 problems. For classification of V1 neurons as excited by or inhibited by AuC stimulation, we split the population of neurons into two groups. Those with a response greater than 0 were included in the excited-by group, and those with a response less than 0 were included in the inhibited-by group (Fig. 3e and Extended Data Fig. 5d–h). For running speed matching (Extended Data Fig. 3b,c), an iterative resampling procedure was used: the fastest and slowest trials were successively removed in the stimulus conditions with higher and lower average running speeds, respectively, until average running speed in the condition with the initially higher average running speed was lower than in the condition with the initially lower average running speed. For Fig. 3d,e, early in conditioning is day 1 of experiment (first exposure to conditioning stimuli), and late in conditioning is the average of the visual responses on days 3 and 4 of conditioning. For the no-reinforcement paradigm (Extended Data Fig. 1g–i), mice were exposed to two sets of stimuli as in the reinforced experiments, A_a_V_a_ and A_b_V_b_, but, as neither condition was reinforced, visual and auditory cue responses were calculated by averaging across both conditions (A_o_V_o_ is the average of A_a_V_a_ and A_b_V_b_; V_o_ is the average of V_a_ and V_b_; and A_o_ is the average of A_a_ and A_b_).

### Statistics and reproducibility

All statistical analyses were performed in MATLAB using custom-written software. Sample sizes were chosen to match typical numbers used in animal behavioral experiments. All data acquired were included in the analysis, with the exception of one mouse that was removed from Fig. 2g,h owing to technical difficulties displaying stimuli during conditioning. Changes in the number of mice (and neurons) across time points are the result of technical difficulties that prevented the acquisition of data in some mice (Supplementary Table 1). Data were first tested for normality using a Lilliefors test, and, when the null hypothesis could not be rejected (h_o_: data come from a normally distributed population), parametric tests were used. Otherwise, non-parametric tests were used. Paired *t*-tests or rank-sum tests were used for analyses with matched samples. For all unmatched samples, data that failed to reject the h_o_ in the Lilliefors test, unpaired t-tests were used (for example, comparisons of axon responses on different conditioning days). Error shading and bars indicate s.e.m. unless otherwise stated in the figure legends. All statistical tests were two tailed. Scattered data were quantified using correlation coefficients, denoted as *r*, and coefficients of determination were computed by taking the square of *r*. For a summary of all statistical tests used, *n* values and exact *P* values, see Supplementary Table 1. No statistical methods were used to determine sample sizes, but sample sizes were selected based on typical sample sizes used in the field. All imaging and behavioral data were acquired from multiple experimental series. Data were additionally subdivided into multiple smaller groups to ensure that effect directions (for example, activity suppression and response differences) were maintained. All efforts to reproduce our results were successful. C57BL/6J mice were assigned randomly to experimental groups defined by injection location and experimental procedure. PV-Cre mice were assigned to optogentic experiments based on their genotype, and our stimulation protocol included randomization of activation laser and sham stimulations. The experimenter was not blinded to group allocation of mice for two-photon and behavioral data but was blinded to mouse identity and cortical region for quantification of histological analyses.

### Reporting Summary

Further information on research design is available in the Nature Research Reporting Summary linked to this article.

## Online content

Any methods, additional references, Nature Research reporting summaries, source data, extended data, supplementary information, acknowledgements, peer review information; details of author contributions and competing interests; and statements of data and code availability are available at 10.1038/s41593-021-00974-7.

## Supplementary information

Supplementary Information Supplementary Table 1

Reporting Summary

## Data Availability

All raw data necessary to reproduce all figures are available at https://data.fmi.ch/.

## Code availability

All analysis code necessary to reproduce all figures is available at https://data.fmi.ch/. Core analysis and imaging code are available at https://sourceforge.net/projects/iris-scanning/.
