# Supplementary material for: A cortical circuit for audio-visual predictions
Source: Nat Neurosci. 2021 Dec 2;25(1):98–105. doi: 10.1038/s41593-021-00974-7 (PMC8737331; doi:10.1038/s41593-021-00974-7)
Supplement: Supplementary file 1 — Supplementary Table 1 [file 41593_2021_974_MOESM1_ESM.pdf]

nature portfolio

https://doi.org/10.1038/s41593-021-00974-7

Supplementary information

A cortical circuit for audio-visual predictions

In the format provided by the authors and unedited

MAIN:

Figure 1

d **test: rank-sum**, response difference index compared to 0 difference across soma

tp	p	# soma	# mice
1	0.258	1548	10
2	0.183	1548	10
3	1.19E-06	1548	10
4	4.77E-28	1548	10
5	4.93E-15	1341	9

f **test: rank-sum**, # lick events during AaVa compared to Va across mice

tp	p	# mice
1	0.4263	10
2	0.3075	10
3	0.064	10
4	0.0452	10
5	0.0039	9

h **test: rank-sum**, response difference index compared to 0 difference across soma

condition	p	# soma	# mice
AbVa to AaVa	1.49E-16	1341	9
AbVa to Va	0.372	1341	9

Figure 2

f **test: rank-sum**, normalized suppression compared to 0

condition	p	# axons	# mice
pre	0.1784	2927	7
post	1.58E-20	3857	7
tone	2.42E-176	4130	6

h **test: paired t-test**, # visually responsive axons to AaVa compared to Va

tp	p	# axons	# mice
1	0.133	5552	8
2	0.0287	4694	7
3	0.0196	4437	7
4	0.0106	4336	6

i **test: unpaired t-test**, # visually responsive axons on day 1 compared to day 4

plot	p
aud-vis	0.0202
vis only	0.5361

tp	# axons	# mice
1	5552	8
4	4658	7

EXTENDED DATA:

Figure 1

f **test: rank-sum**, response difference index compared to 0 difference across soma

tp	p	soma	mice
1	3.58E-23	1548	10
2	2.42E-31	1548	10
3	7.04E-08	1548	10
4	4.74E-09	1548	10
5	1.57E-15	1341	9

tp	p
3	
i (light)	0.0962
ii (med)	0.0027
iii (drk)	1.67E-08

i **test: rank-sum**, response difference compared to 0 difference across soma

tp	p	soma	mice
1	0.061	496	7
2	0.0069	496	7
3	0.0457	496	7
4	0.0015	496	7
5	7.81E-04	335	5

Figure 2

a **test: paired t-test**, comparison of r^2 on day 1 and day 4 across mice

p	tp	soma	mice
0.0391	1	1548	10
	4	1548	10

Figure 3

b & c **test: rank-sum**, response difference index compared to 0 difference across soma

tp	p	soma	mice
4	5.41E-10	1548	10
5	5.44E-14	1341	9

e **test: rank-sum**, comparison of response difference index between AaVa and AaVb

tp	p	soma	mice
4	2.52E-04	1341	9

Figure 5

a **test: correlation coefficients**, null: no relationship exists between variables

p	r	soma	mice
0.71	-0.016	563	5

c **test: correlation coefficients**, null: no relationship exists between variables

p	r	soma	mice
6.51E-252	0.9334	563	5

f **test: rank-sum**, comparison of response difference indices between excited and inhibited soma

tp	p	soma	# excited	# inhibited	mice
5	8.67E-13	1341	927	414	9

h **test: rank-sum**, comparison of response difference indices between excited and inhibited soma

tp	p	soma	# excited	# inhibited	mice
5	1.10E-08	1341	927	414	9

Figure 6

b **test: rank-sum**, reinforced: pre vs post conditioning (p. vs p.)
unreinforced: reinforced vs unreinforced post cond. (r. vs u.)

stimulus	p	mice
aud (p. vs p.)	4.38E-07	5
vis (p. vs p.)	7.71E-13	5
opto (p. vs p.)	0.0169	5
opto (r. vs u.)	0.0035	7
