# Supplementary material for: A cortical circuit for audio-visual predictions
Source: Nat Neurosci. 2021 Dec 2;25(1):98–105. doi: 10.1038/s41593-021-00974-7 (PMC8737331; doi:10.1038/s41593-021-00974-7)
Supplement: Supplementary file 2 — Reporting Summary [file 41593_2021_974_MOESM2_ESM.pdf]

nature portfolio

Corresponding author(s): Aleena R. Garner
Georg B. Keller

Last updated by author(s): Oct 20, 2021

Reporting Summary

Nature Portfolio wishes to improve the reproducibility of the work that we publish. This form provides structure for consistency and transparency in reporting. For further information on Nature Portfolio policies, see our Editorial Policies and the Editorial Policy Checklist.

Statistics

For all statistical analyses, confirm that the following items are present in the figure legend, table legend, main text, or Methods section.

n/a	Confirmed	
☐	☒	The exact sample size (*n*) for each experimental group/condition, given as a discrete number and unit of measurement
☐	☒	A statement on whether measurements were taken from distinct samples or whether the same sample was measured repeatedly
☐	☒	The statistical test(s) used AND whether they are one- or two-sided<br>*Only common tests should be described solely by name; describe more complex techniques in the Methods section.*
☒	☐	A description of all covariates tested
☐	☒	A description of any assumptions or corrections, such as tests of normality and adjustment for multiple comparisons
☐	☒	A full description of the statistical parameters including central tendency (e.g. means) or other basic estimates (e.g. regression coefficient) AND variation (e.g. standard deviation) or associated estimates of uncertainty (e.g. confidence intervals)
☐	☒	For null hypothesis testing, the test statistic (e.g. *F*, *t*, *r*) with confidence intervals, effect sizes, degrees of freedom and *P* value noted<br>*Give P values as exact values whenever suitable.*
☒	☐	For Bayesian analysis, information on the choice of priors and Markov chain Monte Carlo settings
☒	☐	For hierarchical and complex designs, identification of the appropriate level for tests and full reporting of outcomes
☒	☐	Estimates of effect sizes (e.g. Cohen's *d*, Pearson's *r*), indicating how they were calculated

Our web collection on statistics for biologists contains articles on many of the points above.

Software and code

Policy information about availability of computer code

Data collection	2-photon imaging and auxiliary data was collected using custom software written in LabView v3.7. Confocal data was collected using VisiView 64-bit Version 3.3 and Zeiss Zen (blue edition).
Data analysis	Data analysis was performed using custom written MATLAB (Mathworks) code.<br>All analyses code necessary to reproduce all figures is available here: https://data.fmi.ch/.<br>Core analysis and imaging code is available here: https://sourceforge.net/projects/iris-scanning/

For manuscripts utilizing custom algorithms or software that are central to the research but not yet described in published literature, software must be made available to editors and reviewers. We strongly encourage code deposition in a community repository (e.g. GitHub). See the Nature Portfolio guidelines for submitting code & software for further information.

Data

Policy information about availability of data

All manuscripts must include a data availability statement. This statement should provide the following information, where applicable:
- Accession codes, unique identifiers, or web links for publicly available datasets
- A description of any restrictions on data availability
- For clinical datasets or third party data, please ensure that the statement adheres to our policy

All raw data necessary to reproduce all figures is available here: https://data.fmi.ch/.

Field-specific reporting

Please select the one below that is the best fit for your research. If you are not sure, read the appropriate sections before making your selection.

☒ Life sciences ☐ Behavioural & social sciences ☐ Ecological, evolutionary & environmental sciences

For a reference copy of the document with all sections, see nature.com/documents/nr-reporting-summary-flat.pdf

Life sciences study design

All studies must disclose on these points even when the disclosure is negative.

Sample size	No statistical methods were used to determine sample sizes. Sample sizes were selected based on typical sample sizes used in the field. (e.g. Salay, L.D. et al. 2018. A midline thalamic circuit determines reactions to visual threat. Nature (557):183-9; Peron, S. et al. 2020. Recurrent interactions in local cortical circuits. Nature (579):256-9). We aimed to minimize the number of animals sacrificed while using enough to demonstrate robustness of any effects (no effects or effect sizes were assumed apriori).
Data exclusions	No acquired data was excluded with the exception of 1 mouse, removed from Fig 2 g and h, due to technical difficulties displaying stimuli during conditioning.
Replication	All imaging and behavioral data were acquired from a minimum of 2 up to approximately 15 independently performed experimental series. Data was additionally subdivided into random smaller groups during analysis to ensure effect directions (e.g. activity suppression, response differences) were maintained. All efforts to reproduce our results were successful.
Randomization	C57BL/6J mice were assigned randomly to experimental groups defined by injection location and experimental procedure. PV-cre mice were assigned to optogentic experiments based on their genotype and our stimulation protocol included randomization of activation laser and sham stimulations.
Blinding	The experimenter was not blind to group allocation of mice for 2-photon and behavioral data acquisition as no effects were apriori assumed and with the exception of one analysis (Ext. Data Fig. 6), different groups of mice (grouped by injection location, stimulus set, or reinforcement condition) were used to address different biological questions and were not directly compared. For Ext. Data Fig 6, data acquisition was identical except for reinforcements and an identical quantitative analysis was performed on both reinforced and unreinforced group datasets. No apriori assumptions were made about possible effects or effect sizes. For histology and quantification of histological data the experimenter was blinded to mouse identity and cortical region. Acquisition of confocal data could not be completely blinded, however mice were organized by number assignment, which did not provide any information about group allocation, and acquisition settings were identical for all mice used in quantitative anaylses.

Reporting for specific materials, systems and methods

We require information from authors about some types of materials, experimental systems and methods used in many studies. Here, indicate whether each material, system or method listed is relevant to your study. If you are not sure if a list item applies to your research, read the appropriate section before selecting a response.

Materials & experimental systems

n/a	Involved in the study	
☒	☐	Antibodies
☒	☐	Eukaryotic cell lines
☒	☐	Palaeontology and archaeology
☐	☒	Animals and other organisms
☒	☐	Human research participants
☒	☐	Clinical data
☒	☐	Dual use research of concern

Methods

n/a	Involved in the study	
☒	☐	ChIP-seq
☒	☐	Flow cytometry
☒	☐	MRI-based neuroimaging

Animals and other organisms

Policy information about studies involving animals; ARRIVE guidelines recommended for reporting animal research

Laboratory animals	Female and male C57BL/6 and PV-cre mice between 3 and 4 months of age were used.
Wild animals	No wild animals were used.
Field-collected samples	The study did not involve samples collected from the field.
Ethics oversight	All animal procedures were approved by and carried out in accordance with guidelines of the Veterinary Department of the Canton Basel-Stadt, Switzerland.

Note that full information on the approval of the study protocol must also be provided in the manuscript.
